# Antimicrobial and antioxidant potentials of non-cytotoxic extracts of corticolous lichens sampled in Armenia

**DOI:** 10.1186/s13568-021-01271-z

**Published:** 2021-07-29

**Authors:** Razmik Sargsyan, Arsen Gasparyan, Gohar Tadevosyan, Hovik Panosyan

**Affiliations:** 1 Department of Biochemistry, Microbiology and Biotechnology, Yerevan State University, Alex Manoogian 1, 0025 Yerevan, Armenia; 2 Lichen Research and Conservation Group, Takhtajyan Institute of Botany of National Academy of Sciences of the Republic of Armenia, Hrachya Acharyan 1, 0063 Yerevan, Armenia; 3 Institute of Molecular Biology, NAS of Armenia, Ezras Hasratyan 7, 0014 Yerevan, Armenia

**Keywords:** Lichens, Crude extracts, Antimicrobial activity, Antioxidant activity, Cytotoxic activity

## Abstract

Due to wide range of secondary metabolites, lichens were used from antiquity as sources of colorants, perfumes and medicaments. This research focuses on exploring the antioxidant, antimicrobial and cytotoxic activities of methanol, ethanol, acetone extracts and aqueous infusions of corticolous lichens sampled from Armenia. Methanol, ethanol and acetone extracts from all tested lichens were active against Gram-positive bacterial strains. The most effective solvent to retrieve antimicrobial compounds was methanol. Aqueous infusions of tested lichens didn’t show any significant antibacterial and antifungal activity. The highest antimicrobial activity was observed for methanol extract of *Ramalina sinensis*. The minimum inhibitory concentration of methanol extract of *Ramalina sinensis* were 0.9–1.8 mg mL^− 1^. *Pseudevernia furfuracea* demonstrated antifungal activity (Ø 12 mm). Methanol extract of *Parmelia sulcata* demonstrated largest 1,1-diphenyl-2-picryl-hydrazil (DPPH) radical scavenging activity (71 %). The cytotoxicity was measured on human HeLa (cervical carcinoma) cell lines using microculture tetrazolium test assay. The IC_50_ values estimated for methanol extracts of *Peltigera praetextata, Evernia prunastri, Ramalina sinensis* and *Ramalina farinacea* species in HeLa cell line were within 1.8–2.8 mg mL^− 1^ and considered as non-cytotoxic. Obtained results suggest that studied lichens can be prospective in biotechnologies as alternative sources of antimicrobial and antioxidant substances.

## Introduction

About 20,000 species of lichens growing on wide variety of substrates like rocks, walls, exposed soil surfaces and as epiphytes on the bark of trees and leaves have been recorded worldwide (Ellis 2012). They are well adapted to survive in various geographical zones, from sea level to high elevations and from equator to polar regions. They are composite organisms consisting fungi as mycobiont and photosynthetic green algae and/or cyanobacteria as photobiont. Recently it was shown that besides cyanobacteria other bacteria also exist in lichen thalli and take part in mutualistic relationship (Bates et al. 2011; Aschenbrenner et al. 2016; Pankratov et al. 2017). Due to this multiparty mutualism they adapted to survive even in extreme environments characterized by high or low temperatures, periodic desiccation, high levels of UV radiation and salinity. To withstand extreme conditions, lichens synthesized metabolites (e.g., UV screens, cryoprotectants, osmolytes) which are valuable sources to develop new biotechnologies (Suzuki et al. 2016). Ability to produce wide range of unique chemical compounds approves usage of lichens from ancient times as sources of colorants, cosmetics and remedies (Suzuki et al. 2016; Ranković 2015; Calcott et al. 2018). For example, *Parmelia sulcata* have been used to treat diseases of respiratory system, while *Xanthoria parietina* and *Letharia vulpina* were used against to cure jaundice and gastrointestinal disorders, respectively (Ranković et al. 2011; Crawford 2015).

To date more than 800 secondary metabolites have benn identified for lichens. The continuing trends in compounds isolated from lichens approved their importance as a source of new natural products (Ranković 2015; Calcott et al. 2018; Oksanen 2006). Long time lichens were out of attention by pharmaceutical industry reasons of which were their slow-growing nature and difficulties to cultivate in laboratory conditions (Calcott et al. 2018; Yamamoto et al. 1998). For the same reason it is difficult to obtain pure lichen metabolites in needful quantity for checking out their biological activities (Ranković 2015; Calcott et al.  2018; Shrestha and Clair 2013).

Many species of lichens in form of infusions, tinctures and different extracts have been historically used in folk medicine of many countries (Crawford 2015). During the last decades, pharmaceutical potential (i.e., antifungal, antibacterial, antiviral, antitumor, cytotoxic, analgesic, antipyretic properties) of lichens sampled from different regions of the glob has been investigated (Ranković 2015; Boustie and Grube 2005; Shukla et al. 2010; Verma and Behera 2015). Despite its small territory Armenia is a crossroad for variety of rare lichen species (Gasparyan and Sipman 2013; Gasparyan et al. 2015), biodiversity and biotechnological potential of which, still remains unexplored.

Armenia is a South-Caucasian landlocked mountainous country with climate contradictions. Diverse of bio-geographical and climatic conditions, well-defined vertical zonation, as well as active tectonic processes in Armenia have contributed to the formation of ecosystems with rich biodiversity and endemic species. In this context Armenia is a crossroad also for variety of rare and still unexplored lichens’ species (Gasparyan et al. 2015).

Continuous and uncontrolled use of synthetic medicaments often causes numerous side effects. This evidence forces scientists to look for new preparations of natural origin. Lichens as alternative sources can be used to search of new bioactive substances (Ranković 2015; Calcott et al. 2018). Since biotechnological potential of lichens distributed on the territory of Armenia still remains unexplored, we aimed to study of bioactivity of the aqueous and different alcoholic extracts of the corticolous lichens sampled from Armenia. In the present study antimicrobial, antioxidant and cytotoxic activities of crude methanol, ethanol and acetone extracts and aqueous infusions from* R. sinensis*,* R. farinacea*,* F. caperata*,* E. prunastri*,* P. subrudecta*,* P. furfuracea*,* P. praetextata* and *P. sulcata* were examined.

## Experimental

### Collection and identification of lichens

Corticolous lichen samples of *Ramalina sinensis* Jatta, *Ramalina farinacea* (L.) Ach., *Flavoparmelia caperata* (L.) Hale, *Evernia prunastri* (L.) Ach., *Punctelia subrudecta* (Nyl.) Krog, *Pseudevernia furfuracea* (L.) Zopf, *Peltigera praetextata* (Sommerf.) Zopf, *Parmelia sulcata* Taylor were collected from the Dilijan National Park (40°39′23″N, 45°01′17″E) and “Zikatar” Environmental Center (41°07′19.02″N, 44°55′32.52″E), which are located in the Tavush province, Armenia. The area mostly covered by temperate deciduous forests predominating by Oriental Beech (*Fagus orientalis*), Hornbeam (*Carpinus* spp.) and Oak (*Quercus* spp.).

Species identification was performed by standard methods and according to the common identification guides and keys (Andreev et al. 2008; Smith 2009; Gasparyan and Sipman 2016). Voucher specimens of all lichens were deposited in the publicly available Herbarium of Yerevan State University (YSU, Yerevan, Armenia) where serial numbers of five lichens were given; *R. sinensis* Jatta (ERHM 11,071), *R. farinacea* (L.) Ach. (ERHM 11,072), *E. prunastri* (L.) Ach. (ERHM 11,073), *P. furfuracea* (L.) Zopf (ERHM 11,074), *P. sulcata* Taylor (ERHM 11,070).

### Preparation of lichen extracts

Lichen thalli (10 g) were grinded using automatic grinder, until obtaining powder-like state. Grinded thalli were drenched with methanol, ethanol and acetone separately at 10:1 solvent-to-sample ratio (v/w). The mixtures with methanol, ethanol and acetone were left for extraction for 24 h on magnetic stirrer, centrifuged (15 min, 12,000 rpm) and then concentrated under reduced pressure in a rotary evaporator (BOV-50 V vacuum drying oven, Biobase Meihua Trading, China) at 37 °C temperature to dry. Extraction process was repeated three times to retrieve active compounds as much as possible. To obtain aqueous infusions the grinded lichens were dissolved in distilled water, then left for extraction for 24 h on magnetic stirrer under heat conditions not exceeding the boiling point. Aqueous infusions filtered through 0.22 μm sterile filter (Millipore). Dried extracts were weighted and stored at − 18 °C until they were used in the tests. Dimethyl sulfoxide (DMSO) (Sigma-Aldrich) was used to prepare stock solutions. The extracts were diluted by sterile water up to 5 % DMSO for the experiments.

### Antimicrobial activity

The microbial strains used in this study were following: Gram-positive bacteria *Bacillus subtilis* WT-A1, *Staphylococcus aureus* MDC 5233, Gram-negative bacteria *Escherichia coli* VKPM-M17, *Pseudomonas aeruginosa* GRP3 and *Salmonella typhimurium* MDC 1754 and a yeast *Candida albicans* WT-174. Microbial strains were from microbial culture collection maintained by the Department of Biochemistry, Microbiology and Biotechnology, YSU.

Agar disc diffusion method was used to evaluate antimicrobial activity of lichens. The bacterial strains were inoculated in Müller-Hinton broth (MHB) and incubated overnight. Bacteria were sub-cultured in MHB liquid medium at 37 °C to OD600 = 0.2. Then 100 µL of inoculum was spread on a Petri dish with Müller-Hinton agar (MHA). Yeasts were inoculated in Sabourad dextrose (SD) broth and incubated overnight. After incubation 100 µL of culture was spread on SD agar. Sterilized Whatman filter paper discs (5 mm diameter) were infiltrated by 5 µL (500 µg mL^− 1^) of extracts and placed on MHA or SD agar plates containing appropriate microbial strain. Diameter of inhibition zones (IZ) formed around discs after incubation at 37 °C for 24 h was measured. The experiments were conducted at least thrice and the average of three measurements was accepted as an index of antibacterial activity. As positive controls gentamicin (10 µg mL^− 1^) (for bacteria) and nystatin 20 µg mL^− 1^ (for yeast) were used; while 5 % DMSO was used as a negative control.

Broth microdilution method was applied to determine minimum inhibitory concentration (MIC) (Wiegand et al. 2008). A series of dilutions ranging from 0.9 to 7.5 mg mL^− 1^ for extracts were used in the experiment. The highest dilution of samples without visible growth after 24 h incubation at 37 °C was considered as MIC. To check sterility of crude extracts all its dilutions were cultured in agar media. The minimum bactericidal/fungicidal concentration (MBC/MFC) values was determined by sub-culturing samples from the tubes with concentrations above the MIC on new plates with MHA and SD agar for bacteria and yeast, respectively. The values obtained were the average data of experiments performed at least three time.

### Antioxidant activity

Free radical scavenging method based on 1,1-diphenyl-2-picryl-hydrazil (DPPH) was used to measure antioxidant activity of lichen extracts (Molyneux 2004). The reactive solution contained 1 mg mL^− 1^ ethanol extract, to which was added 0.1 mM DPPH. The absorbance was measured spectrophotometerically (λ 517 nm). As positive control ascorbic acid was used. In negative control, the extract was replaced by ethanol. The following equation was used to evaluate radical scavenging activity (RSA): RSA (%) = [(A_0_ − A_1_)/A_0_] × 100, where A_0_ absorbance of the negative control, A_1_ absorbance of reaction mixture or standard (Gao et al. 2000).

### Determination of total phenolic compounds (TPC)

TPC was measured by the Folin-Ciocalteu method (Meda et al. 2005). 1 mL of methanol extracts of 1 mg mL^− 1^ aliquots were mixed with 5 mL of Folin-Ciocalteu reagent (diluted 1:10) and 15 mL of 20 % (w/v) sodium carbonate solution. The mixture was incubated at room temperature in the dark for 1 h and absorbance was measured at 765 nm. TPC was calculated as gallic acid equivalents (GAE) per 100 g of lichen thalli (mg GAE/100 g).

### Determination of total flavonoid content (TFC)

TFC was determined by the Dowd method (Meda et al. 2005). The mixture containing 2 mL of methanol extracts (1 mg mL^− 1^) and 2 mL of methanol solution of aluminum trichloride (2 %, v/w) was incubated at room temperature for 30 min. The absorbance was measured at 420 nm. Negative control, without extract was used as a blank. TFC was determined as microgram of catechin equivalent by using an equation that was obtained from standard catechin graph. The result was expressed as mg of catechin equivalents per 100 g of lichen thalli (mg CE/100 g).

### Cytotoxic activity

The human HeLa (cervical carcinoma cell line) cells from the European Collection of Authenticated Cell Cultures, UK were used in experiments. The cancer cell lines were routinely maintained and cultivated in Dulbecco’s Modified Eagle’s medium (Sigma Aldrich, Germany) supplemented with 10 % fetal bovine serum (HyClone, UK), 2 mM l-glutamine (Sigma Aldrich, Germany), 100 IU mL^− 1^ penicillin (Sigma Aldrich, Germany) and 100 µg mL^− 1^ streptomycin (Sigma Aldrich, Germany). Cells were incubated in humidified atmosphere containing of 95 % air and 5 % CO_2_ at 37 °C.

Microculture tetrazolium test (MTT) assay was used to determine the effect of extracts on cancer cell survival (Van de Loosdrecht et al. 1994). The cells were seeded at the density of 0.1 × 10^6^ cell/ mL^− 1^ into 96-well plates (Greiner, Germany). After incubation for 24 h, different concentration of extracts obtained by diluting the stock solution (1:10, 1:50, 1:100, 1:200) were added to the cell cultures. The stock solution concentrations were for *R. sinensis* 177 mg mL^− 1^, for *R. farinacea* 391 mg mL^− 1^, for *E. prunastri* 600 mg mL^− 1^, and for *P. praetextata* 328 mg mL^− 1^. The cells treated with DMSO (Sigma Aldrich, Germany) were used as vehicle control. After further incubation for 48 h, the MTT dye (Sigma Aldrich, Germany) was added to each well (500 µg mL^− 1^ final concentration) and incubated for 4 h at 37 °C. Then the supernatant was removed and 100 µL of DMSO was added. Enzyme-linked immunosorbent assay (ELISA) plate reader (Human Reader HS, Germany) was used to measure the absorbance (λ 570 nm). Cell viability was expressed as a percentage of the negative control (cell cultures with no treatment). To reveal the cytotoxicity of the extract’s doses inducing 50 % inhibition of cell viability (IC_50_ value) were determined.

### Data processing

GraphPad Prism 5.01 (GraphPad Software, USA) was used to perform data analysis. All experiments were conducted in triplicates. Values were expressed as means ± standard error (SE). Data were analyzed by repeated measures ANOVA. Dunn’s post-hoc test was used to determine differences between groups. p < 0.05 values were considered as the statistically significant.

## Results

### Antibacterial activity

Results of antimicrobial activity of alcoholic extracts and aqueous infusions of tested lichens against tested bacteria and yeast are summarized in the Table 1. Diameters of IZ around used paper disks were measured to evaluate antimicrobial activity qualitatively. Aqueous infusions of all tested lichens did not demonstrate any significant antibacterial and anticandidal activity. Methanol, ethanol and acetone extracts from all tested lichens were demonstrated antibacterial activity against Gram-positive bacterial strains i.e., *B. subtilis* and *S. aureus*, while they were not able to inhibit the growth of tested Gram-negative bacteria.

**Table 1:** Disc diffusion inhibition zones (IZ) minimum inhibitory concentrations (MIC) and minimum bactericidal/fungicidal concentrations (MBC/MFC) of tested lichen crude extracts

Lichens species	Crude extracts^a^	Test microbes, IZ [Ø (mm)], MIC (mg mL^− 1^) and MBC/MFC (mg mL^− 1^)																	
		*B. subtilis*			*S. aureus*			*E. coli*			*P. aeruginosa*			*S. typhymurium*			*C. albicans*		
		IZ	MIC	MBC	IZ	MIC	MBC	IZ	MIC	MBC	IZ	MIC	MBC	IZ	MIC	MBC	IZ	MIC	MFC
*R. sinensis*	M	25 ± 0.5	0.9	0.9	15 ± 0.5	1.8	1.8	1.5 ± 0.3	> 7.5	> 7.5	2.0 ± 0.3	> 7.5	> 7.5	1.5 ± 0.3	> 7.5	> 7.5	0	nd	nd
	E	23 ± 0.5	nd	nd	12 ± 0.5	nd	nd	1.0 ± 0.3	nd	nd	1.2 ± 0.3	nd	nd	1.2 ± 0.3	nd	nd	0	nd	nd
	A	21 ± 0.5	nd	nd	13 ± 0.5	nd	nd	1.0 ± 0.3	nd	nd	1.0 ± 0.3	nd	nd	1.0 ± 0.3	nd	nd	0	nd	nd
	W	0	nd	nd	0	nd	nd	0	nd	nd	0	nd	nd	0	nd	nd	0	nd	nd
*R. farinacea*	M	23 ± 0.5	1.8	1.8	15 ± 0.5	3.75	3.75	1.2 ± 0.3	> 7.5	> 7.5	1.5 ± 0.3	> 7.5	> 7.5	1.0 ± 0.3	> 7.5	> 7.5	0	nd	nd
	E	21 ± 0.5	nd	nd	11 ± 0.5	nd	nd	1.0 ± 0.3	nd	nd	< 1	nd	nd	1.2 ± 0.3	nd	nd	0	nd	nd
	A	19 ± 0.5	nd	nd	10 ± 0.5	nd	nd	1.0 ± 0.3	nd	nd	< 1	nd	nd	1.0 ± 0.3	nd	nd	0	nd	nd
	W	0	nd	nd	0	nd	nd	0	nd	nd	0	nd	nd	0	nd	nd	0	nd	nd
*E. prunastri*	M	19 ± 0.5	3.75	3.75	17 ± 0.5	3.75	3.75	< 1	nd	nd	1.3 ± 0.3	> 7.5	> 7.5	< 1	> 7.5	> 7.5	0	nd	nd
	E	15 ± 0.5	nd	nd	14 ± 0.5	nd	nd	< 1	nd	nd	1.1 ± 0.3	nd	nd	< 1	nd	nd	0	nd	nd
	A	10 ± 0.5	nd	nd	9 ± 0.5	nd	nd	< 1	nd	nd	1.0 ± 0.3	nd	nd	< 1	nd	nd	0	nd	nd
	W	0	nd	nd	0	nd	nd	0	nd	nd	0	nd	nd	0	nd	nd	0	nd	nd
*F. caperata*	M	17 ± 0.5	7.5	> 7.5	19 ± 0.5	3.75	3.75	< 1	nd	nd	1.0 ± 0.3	-	-	1.8 ± 0.3	> 7.5	> 7.5	0	nd	nd
	E	15 ± 0.5	nd	nd	16 ± 0.5	nd	nd	0	nd	nd	< 1	nd	nd	1.2 ± 0.3	nd	nd	0	nd	nd
	A	12 ± 0.5	nd	nd	14 ± 0.5	nd	nd	0	nd	nd	< 1	nd	nd	1.0 ± 0.3	nd	nd	0	nd	nd
	W	0	nd	nd	0	nd	nd	0	nd	nd	0	nd	nd	0	nd	nd	0	nd	nd
*P. subrudecta*	M	15 ± 0.5	7.5	> 7.5	10 ± 0.5	> 7.5	> 7.5	1.0 ± 0.3	> 7.5	> 7.5	1.6 ± 0.3	> 7.5	> 7.5	< 1	nd	nd	0	nd	nd
	E	10 ± 0.5	nd	nd	8 ± 0.5	nd	nd	1.3 ± 0.3	nd	nd	1.2 ± 0.3	nd	nd	< 1	nd	nd	0	nd	nd
	A	9 ± 0.5	nd	nd	7 ± 0.5	nd	nd	1.5 ± 0.3	nd	nd	1.3 ± 0.3	nd	nd	< 1	nd	nd	0	nd	nd
	W	0	nd	nd	0	nd	nd	0	nd	nd	0	nd	nd	0	nd	nd	0	nd	nd
*P. furfuracea*	M	15 ± 0.5	7.5	> 7.5	8 ± 0.5	> 7.5	> 7.5	< 1	nd	nd	1.5 ± 0.3	> 7.5	> 7.5	1.2 ± 0.3	> 7.5	> 7.5	12 ± 0.5	3.75	> 7.5
	E	7 ± 0.5	nd	nd	6 ± 0.5	nd	nd	< 1	nd	nd	1.0 ± 0.3	nd	nd	1.2 ± 0.3	nd	nd	0	nd	nd
	A	9 ± 0.5	nd	nd	3 ± 0.5	nd	nd	< 1	nd	nd	1.2 ± 0.3	nd	nd	1.0 ± 0.3	nd	nd	0	nd	nd
	W	0	nd	nd	0	nd	nd	0	nd	nd	0	nd	nd	0	nd	nd	0	nd	nd
*P. sulcata*	M	13 ± 0.5	7.5	> 7.5	9 ± 0.5	7.5	> 7.5	< 1	nd	nd	1.4 ± 0.3	> 7.5	> 7.5	1.2 ± 0.3	> 7.5	> 7.5	0	nd	nd
	E	9 ± 0.5	nd	nd	8 ± 0.5	nd	nd	0	nd	nd	1.0 ± 0.3	nd	nd	< 1	nd	nd	0	nd	nd
	A	8 ± 0.5	nd	nd	5 ± 0.5	nd	nd	0	nd	nd	1.0 ± 0.3	nd	nd	< 1	nd	nd	0	nd	nd
	W	0	nd	nd	0	nd	nd	0	nd	nd	0	nd	nd	0	nd	nd	0	nd	nd
*P. praetextata*	M	13 ± 0.5	7.5	> 7.5	10 ± 0.5	7.5	> 7.5	< 1	nd	nd	1.2 ± 0.3	> 7.5	> 7.5	< 1	nd	nd	0	nd	nd
	E	9 ± 0.5	nd	nd	9 ± 0.5	nd	nd	0	nd	nd	1.0 ± 0.3	nd	nd	< 1	nd	nd	0	nd	nd
	A	9 ± 0.5	nd	nd	7 ± 0.5	nd	nd	0	nd	nd	1.0 ± 0.3	nd	nd	< 1	nd	nd	0	nd	nd
	W	0	nd	nd	0	nd	nd	0	nd	nd	0	nd	nd	0	nd	nd	0	nd	nd
PC^b^		32 ± 0.5	0.25	0.5	28 ± 0.5	0.3	0.5	20 ± 0.5	0.5	1.5	25 ± 0.5	0.3	0.5	23 ± 0.5	1	2.5	20 ± 0.5	1.75	4

*nd* not determined

^a^Extracts: M—methanol, E—ethanol, A—acetone, W—water

^b^PC: positive control, for IZ: gentamicin (10 µg mL^− 1^) for bacteria, nystatin 20 µg mL^− 1^ for yeasts; for MIC and MBC/MFC: gentamicin (range 2–0.003 µg mL^− 1^) for bacteria, nystatin (range 4–0.125 µg mL^− 1^) for yeasts

The most antibacterial activity was observed for methanol extracts, which even at low concentrations were able to inhibit all tested Gram-positive bacteria. The methanol and ethanol extracts of *R. sinensis* showed the largest IZs (25 mm and 23 mm, respectively) against the *B. subtilis*, while largest IZs (19 mm and 16 mm, respectively) of the same extracts against *S. aureus* was observed in case of *F. caperata.* The same pattern is observed in case of acetone extracts of the same lichens, where largest IZs against to *B. subtilis* and *S. aureus* were 21 mm and 14 mm, respectively.

Although all alcoholic extracts of the lichen *P. fufuracea* manifested lowest antibacterial activity, it was only lichen, methanol extract of which, demonstrated antifungal activity against *C. albicans* (Ø, 12 mm).

Since the maximum antimicrobial activity by agar disc diffusion tests was found in methanol extracts, antimicrobial activities subsequently were quantitatively evaluated by MICs and MBCs/MFCs values only for methanol extracts. MICs values were determined for each bacterium. Methanol extract from *R. sinensis* was shown relatively high antibacterial effect (MIC 0.9 mg mL^− 1^) at the concentrations used. MIC for *B. subtilis* varied from 0.9 to 7.5 mg mL^− 1^, while MIC for *S. aureus* ranged from 0.9 to more than 7.5 mg mL^− 1^. MBC were determined for *B. subtilis* (in case of *R. sinensis* it was 0.9 mg mL^− 1^) and *S. aureus* (ranged from 1.8 to > 7.5 µg mL^− 1^). MFC value observed only for methanol extract of *P. fufuracea* was > 7.5 µg mL^− 1^.

Gentamycin and nystatin were used as standard antibiotics to compare antimicrobial activities obtained for bacteria and yeast, respectively. The results confirmed that antimicrobial activities were several time higher in case of standard antibiotics. DMSO didn’t show inhibitory effect on the tested organisms.

### Antioxidant activity

**Fig. 1:**
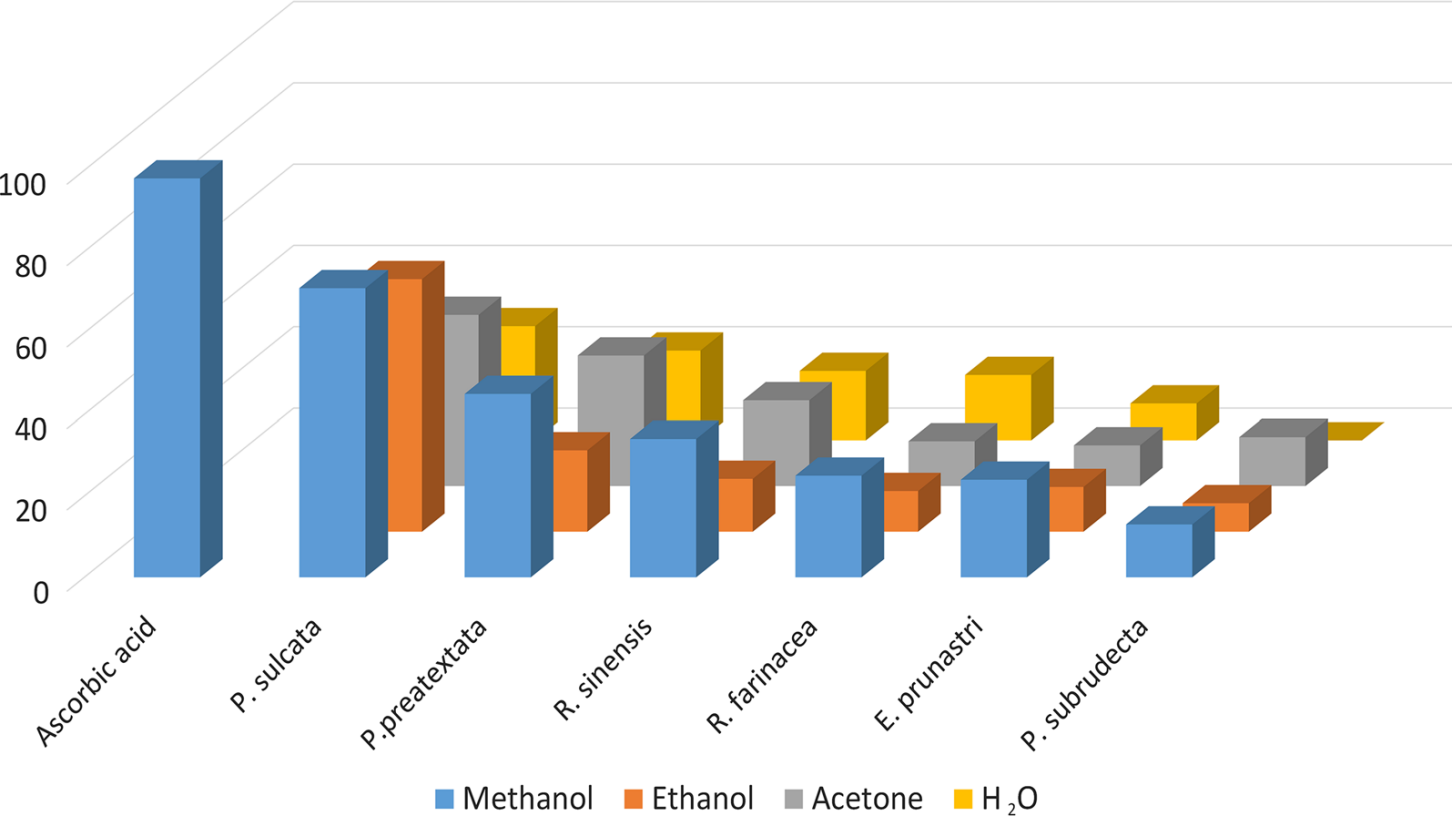
DPPH radical scavenging activity (%) of methanol, ethanol, acetone extracts and aqueous infusion of *P. sulcata R. sinensis P. praetextata R. farinacea, P. subrudecta* and *E. prunastri*. Ascorbic acid was used as positive control

The scavenging activity of DPPH radicals of lichen extracts is shown in Fig. 1. Methanol extracts showed a good radical scavenging activity. The highest activity showed methanol extract of *P. sulcata* with 71 % activity, which was only slightly lower compared with ascorbic acid (96 ± 2%). Methanol extract of *P. preatextata* was also demonstrated promising scavenging activity (44 %). The methanol extract of other tested lichens showed slightly weaker DPPH radical scavenging activities (< 30 %). Relatively higher scavenging activity was observed also of acetone extracts of lichens *P. praetextata* and *R. sinensis.* Ethanol and aqueous extracts were not shown significant scavenging activity of DPPH radicals. Surprisingly, aqueous extracts of *P. praetextata, R. sinensis* and *R. farinacea* have shown more antiradical activity then ethanol extract of the same lichens. These results were somewhat unexpected, since usually the ethanol extracts exhibiting higher radical scavenging abilities.

TPC and TFC of tested methanol extracts are shown in Table 2. Highest phenolic compounds was identified in extract of *P. sulcata* 3811 mg GAE per 100 g lichen dry weight, while extract of *P. subrudecta* showed the lowest content (608 mg GAE per 100 g lichen dry weight). Relatively high phenolic compounds was determined also for *E. prunastri* (3585 mg GAE/100 g).

**Table 2:** TPC and TFC of methanol extracts of lichens

Lichen species	TFC		TPC	
	Present study (mg CE/ 100 g Dw)	Literature data	Present study (mg GAE/ 100 g Dw)	Literature data
*P. sulcate*	700 ± 7.1	9.6 ± 1.09 µg RuE/ mg extract^a^	3811 ± 71.25	25.1 ± 1.11 µg PCE/ mg extract ^a^
*E. prunastri*	373 ± 4.2	20 ± 3 µg QE/ mg extract^b^	3585 ± 69.30	90 ± 3 µg GAE/ mg extract^b^
*P. preatextata*	310 ± 3.7	NA	1648 ± 72.3	109.3 ± 0.9 µg CE/ mg extract^d^
*F. caperata*	567 ± 6.3	27.46 ± 0.78 µg RuE/ mg extract^c^	1522 ± 67.2	90.83 ± 0.98 µg GA/ mg extract^c^
*R. farinacea*	295 ± 2.1	20 ± 3 µg QE/ mg extract^b^	1128 ± 70	75 ± 3 µg GAE/ mg extract^b^
*R. sinensis*	523 ± 5.1	NA	786.5 ± 56	14.7 ± 0.8 µg CE/ mg extract^d^
*P. subrudecta*	222 ± 2.3	NA	608 ± 42.1	NA

*Dw* dry weight,* NA* not availbale,* QE* quercetin,* RuE* rutin equivalent,* PCE* pyrocatechol equivalent

^a^Data from Kosanic et al. (2010)

^b^Data from Aoussar et al. (2021)

^c^Data from Mitrovic et al. (2011)

^d^Data from Luo et al. (2010)

TFC for methanol extracts of *P. sulcata*, *F. caperata* and *R. sinensis* were 700, 567 and 523 mg CE/100 g lichen dry weight, respectively. The methanol extract of *P. sulcata* showed highest TFC among all lichen extracts, the lowest content of flavonoids was observed for P. subrudecta extracts (222 mg CE/100 g lichen dry weight). TPC and TFC of methanol extracts of studied lichen species were compared with those isolated in different part of the world (Table 2).

### Cytotoxic activity

**Fig. 2:**
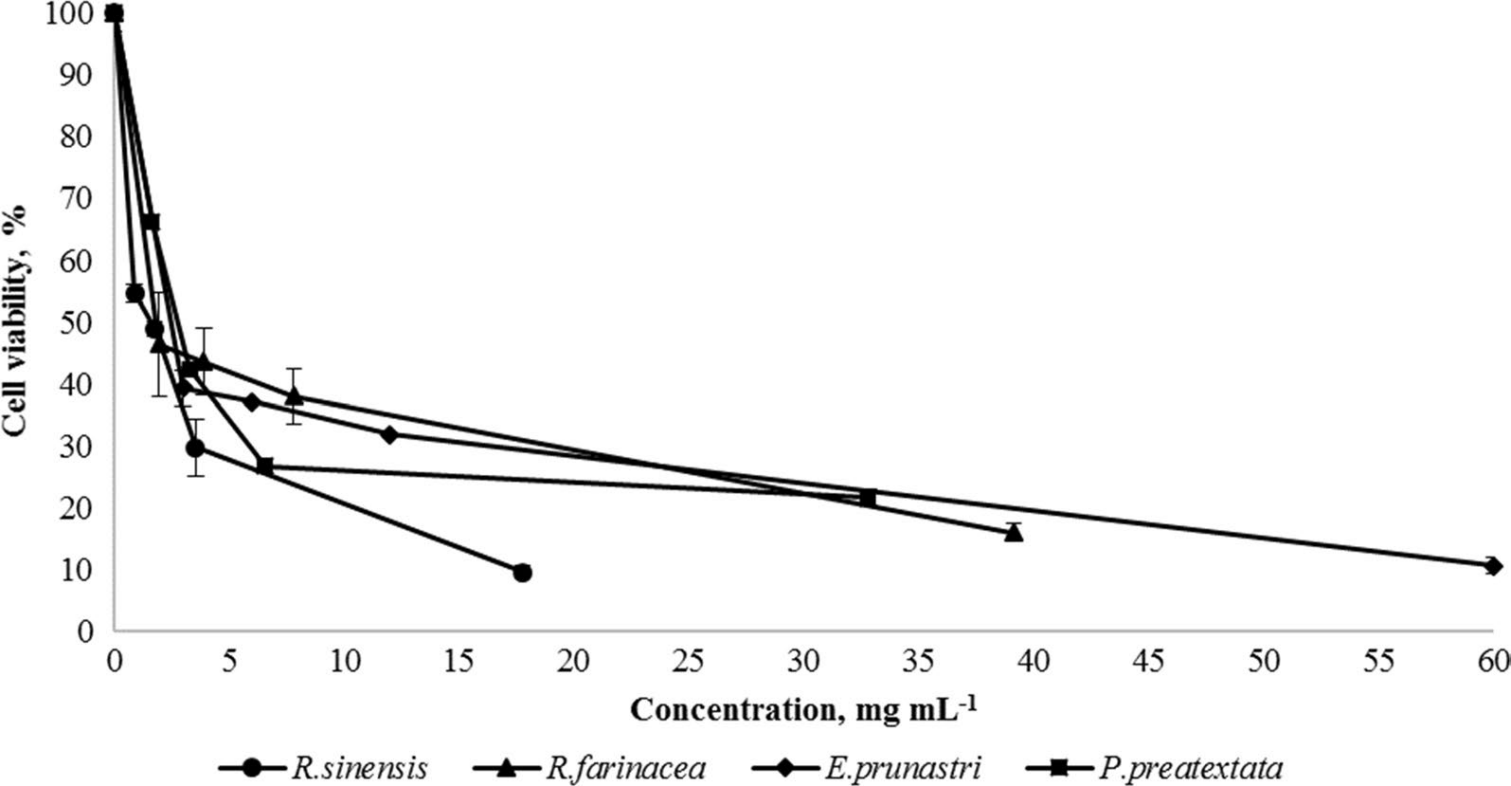
Dose-dependent effects of methanol extracts of* R. sinensis*,* R. farinacea*,* P. praetextata* and *E. prunastri* on viability of HeLa cells

The statistically significant and dose-dependent decrease in cell viability was shown for methanol extracts of all tested lichens started from lower tested concentrations (Fig. 2). The IC_50_ values of *R. sinensis* and *R. farinacea* extracts were almost similar 1.8 ± 0.1 mg mL^− 1^ and 1.75 ± 0.4 mg mL^− 1^, respectively. However, at higher tested concentrations, the cytotoxic profile of mentioned extracts was different, since 10 % of viability was observed at the concentration of 18 mg mL^− 1^ for *R. sinensis* and 40 mg mL^− 1^ for *R. farinacea* extracts. The similar cytotoxic activity was shown for *P. praetextata* and *E. prunastri* extracts at all tested concentrations and the IC_50_ values were 2.8 ± 0.3 mg mL^− 1^ and 2.4 ± 0.2 mg mL^− 1^, respectively.

## Discussion

In our experiments, aqueous infusions of all tested lichens lack of antibacterial and antifungal activities, which coincides with literature data (Kosanic and Rankovic 2014). Weak activity of aqueous infusions is probably result of insolubility or poor solubility of secondary metabolites in water (Kinoshita et al. 1994). Despite this generally accepted opinion, some researchers have also shown antimicrobial activity of some aqueous extracts of lichens. Thus, Karagouml et al. (2009) showed that aqueous extracts of *R. farinacea* and some species belonging to the genera *Anaptychia*, *Peltigera*, *Umbilicaria*, *Xanthoria* and *Xanthoparmelia* exhibited potent inhibition toward *E. coli*, *B. subtilis* and *S. aureus*. Recently it was shown aqueous extracts from Ecuadorian lichens *Usnea* sp. possessed antibacterial activity against *B. subtilis* (Matvieieva et al. 2015).

In contrast to aqueous extracts, alcoholic extracts of lichens in our experiments demonstrated relatively high antibacterial activity. The quality of the antibacterial effect depended on the species of lichen. Within tested lichen extracts only *R. sinensis* has demonstrated significant bactericidal activity against *B. subtilis*. Probably *R. sinensis* possessed activity against endospores as well. The methanol and ethanol extracts of *R. sinensis* showed the maximum antibacterial activity. The methanol extract of *F. caperata* was active against *S. aureus*.

Only static activity was observed against other tested microbes. MBC/MFC almost in all cases were higher than respective MIC values. According to the results obtained Gram-positive bacteria were more sensitive against the crude extracts of tested lichens. Such selective inhibition by extracts can be explained by composition and structural peculiarities of bacterial cell walls. Gram-positive bacterial cell walls endowed with higher permeability than Gram-negative bacterial ones (Kosanic and Rankovic 2014).

Antimicrobial features of different extracts of lichen species belonging to genus *Ramalina* were investigated by other researchers too. Thus, ethanol extract of *R. farinacea* sampled from New Zealand showed inhibitory effect toward bacilli and some Gram-negative bacteria (Esimone and Adikwn 1999). Both tested Gram-positive and Gram-negative bacteria were sensitive against ethanol extract of *R. farinacea* sampled from Turkey (Karagouml et al. 2009). Lichen species sampled from Antarctic also demonstrated high antibacterial properties against to *S. aureus* and *B. subtilis* (Bhattarai et al. 2008; Mitrovic et al. 2011) also reported about strong inhibitory effect of methanol extracts of *P. sulcata*, *F. caperata* and *E. prunastri* against mainly Gram-positive bacteria. In general results obtained in this study are in agreement with literature data and confirmed high antibacterial activity of tested lichens.

Among tested lichens, *Pseudevernia furfuracea* was the single species demonstrating the antifungal activity. There are some reports revealing high resistance of fungi against antimicrobial agents of lichen origin. Presumably it depends on specific composition and permeability of its cell wall (Kosanic and Rankovic 2014).

In contrast to our results, it was shown that aqueous and different polar and nonpolar extracts of many lichens also demonstrated antifungal activity. For instance, Karabuluti and Ozturk (2015) reported that some extracts of *E. prunastri*, *P. sulcata* and *P. furfuracea* demonstrated significant antifungal activity against species of genera *Aspergillus*, *Botrytis*, *Fusarium*, *Macrophomina*, *Penicillium* and *Rhizoctonia*. Using disc diffusion method Türkan et al. (2013) was shown anticandidal activity of acetone and chloroform extracts of *P. furfuracea*. Similar investigation carried out using nonpolar fractions of *P. furfuracea* exhibited significant antifungal activity especially against (Güvenç et al. 2012).

It was shown earlier that lichen thalli comprise numerous secondary metabolites with antibacterial and antifungal activity (Ranković 2015; Calcott et al. 2018; Crawford 2015; Boustie and Grube 2005; Verma and Behera 2015). The type of extracting solvent also has a decisive significance. Considering this we aimed also evaluate solvents efficiency to extract bioactive compounds from lichen thalli. As mentioned above methanol extracts demonstrated the highest antimicrobial activities. Second strongest antimicrobial activities were recorded in case of ethanol extracts, followed by acetone. Thus, we assumed that in our investigation methanol was efficient solvent to extract phenolic and/or other compounds with antimicrobial activity.

Azmir et al. (2013) evaluated the impact of solvent on the extraction process of phytochemicals. It was shown that based on the polarity of the solvent, particular compounds may be extracted (Table 3). Hereby we can conclude that highest activity of methanol extracts is correlating with the fact that, using methanol as a solvent derives abundant variety of bioactive compounds.

**Table 3:** Example of some extracted bioactive compounds by different solvents. Adapted from Azmir et al. (2013)

Solvent	Water	Ethanol	Methanol	Acetone
Bioactive compound	Anthocyanins tannins Saponins terpenoids	Tannins Polyphenols flavonol Terpenoids alkaloids	Anthocyanin terpenoidssaponins Tannins Flavones polyphenols Chloroform	Flavonoids

The tested lichen methanol extracts also expose relatively strong antioxidant activities against DPPH radical *in vivo.* The strong antioxidant activity is probably connected with the substances extracted by methanol (Azmir et al. 2013). It is distinctive that water extracts of *P. praetextata, R. sinensis, R. farinacea* and *E. prunastri* also derive relatively strong antioxidant activity. Moreover, the antioxidant activity of aqueous infusions of *P. praetextata, R. sinensis* and *R. farinacea* exceeds the activity of ethanol extracts of the same species. The scavenging activity is possible associated with secondary metabolites which are unique for that species and type of solvent. In this case, as it was mentioned the most efficient solvent was methanol.

Kumar et al. (2014) reported existence of correlation between some secondary metabolites (mainly phenols) in lichen thalli and its antioxidant properties. Correlation between phenolic and flavonoid compounds of the tested extracts and free radical scavenging activity were shown in our study too. The tested methanol extracts of *P. sulcata* exhibited the highest radical scavenging activity with the greatest amount of phenolic and flavonoid contents. However, recently some deviations from this pattern have been also shown (Odabasoglu et al. 2005). This evidence allowed to assume that antioxidant activity can be conditioned other, non-phenol components. Gülcin et al. (2002) showed strong antioxidant activity of aqueous extracts of *Cetraria islandica*. Stanly et al. (2011), studying some Malaysian lichens found contradiction between antioxidant activity and total phenol content. In contrast to this, methanol extracts of the lichen species *P. sulcata, F. caperata, E. prunastri, Hypogymnia physodes* and *Cladonia foliacea* collected from southeast of Serbia demonstrated high antioxidant activities (Mitrovic et al. 2011). In our studies, we also clearly showed that not only alcoholic solvents (which usually extracts phenolic compounds), but aqueous extracts also demonstrated antioxidant activity. Moreover, methanol extract of *P. preatextata* demonstrated relatively high DPPH radical radical scavenging activity (44 %), but TPC and TFC were low. Despite of high flavonoid and phenolic content the radical scavenging activity of methanol extract of *E. prunastri* was very low (Table 2; Fig. 1).

There are other reports regarding to antioxidant effect of lichens. Thus, Ranković et al. (2011) showed free radical scavenging activity (94.7 % inhibition) for acetone extract of *Lecanora atra*. In this study, the highest activity was observed for methanol extract of *P. sulcata* with 71 % activity. For comparison, it should be noted that methanol extract of another representative of the genus *Parmelia* (*P. saxatilis*) had free radical-scavenging activity with 55.3 % inhibition (Kosanić et al. 2012a). To our knowledge it is the first report about high antioxidant activity observed for methanol extracts of *P. sulcata.*

It was established that some lichen secondary metabolites (usnic acid, lecanoric acid, lobaric acid, evernic acid, vulpinic acid and so on) have cytotoxic properties (Shrestha and Clair 2013). Cytocidal effect of mentioned metabolites displays by cell cycle arrest, apoptosis, necrosis, and inhibition of angiogenesis (Brisdelli et al. 2013).

Earlier the cytotoxicity of *E. prunastri* extracts was analyzed in different cell lines. The weak cytotoxic effect (IC_50_ = 120.89 µg mL^− 1^) was shown for acetone extract of *E. prunastri* in FemX and LS 174 cell lines lines (Kosanić et al. 2012b). Non-cytotoxic properties of *E. prunastri* methanol extract was revealed on colon cancer adenocarcinoma cell line HCT-116 (IC_50_ = 295.64 µg mL^− 1^) (Mitrovic et al. 2011). A crude extract of *Xanthoria parietina* significantly inhibited growth of Murine myeloma P3 × 63-Ag8.653 cells (Triggiani et al. 2009). Only few publications are available demonstrating anticancer activity of lichen extracts. Ari et al. (2015) reported significant anticancer effect (IC_50_ values 16.5 µg mL^− 1^) for methanol extract of *P. sulcata* against Human Breast cancer cell lines MDAMB-231. Viable cell number of Human colon cancer cell (HT-29) line was decreased after treatment them by acetone and methanol extracts of *Lethariella zahlbruckneri* (Ren et al. 2009).

In present study, the IC_50_ values of methanol extracts of studied lichens were in the range of 1.8–2.8 mg mL^− 1^. According to the American National Cancer Institute, a crude extract is considered as active for an IC_50_ < 30 μg mL^−1^ in the preliminary assay (Suffness and Pezzuto 1990). Following this criterion, the methanol extracts of studied lichen species (*P. praetextata, E. prunastri, R. sinensis, R. farinacea*) cannot be considered as cytotoxic. Since compounds possessing potential antimicrobial and antioxidant activities may not be useful in pharmacological preparations if they possess cytotoxic effect, the non-cytotoxic profile of extracts studied in our work proves their safety and extracts can be recommended for further studies. The obtained results stated strong antioxidant, antimicrobial activity and non-cytotoxic profile of tested lichen extracts. Lichens stand as organisms with high biotechnological potential, which was proven before by various authors, but was reported for the first time for lichens distributed on the territory of Armenia.

## Data Availability

Lichens are available in the Herbarium of Yerevan State University (Yerevan, Armenia). Materials and data of this study are available upon request.
